# Major Depressive Disorder and Kappa Opioid Receptor Antagonists

**Published:** 2016

**Authors:** Wei Li, Huijiao Sun, Hao Chen, Xicheng Yang, Li Xiao, Renyu Liu, Liming Shao, Zhuibai Qiu

**Affiliations:** 1 Department of Medicinal Chemistry, School of Pharmacy, Fudan University; 2 Department of Anesthesiology and Critical Care, Perelman School of Medicine, University of Pennsylvania

**Keywords:** Major depressive disorder, Kappa opioid receptor, Antagonists

## Abstract

Major depressive disorder (MDD) is a common psychiatric disease worldwide. The clinical use of tricyclic antidepressants (TCAs), monoamine oxidase inhibitors (MAOIs) and selective serotonin reuptake inhibitors (SSRIs)/serotonin–norepinephrine reuptake inhibitor (SNRIs) for this condition have been widely accepted, but they were challenged by unacceptable side-effects, potential drug-drug interactions (DDIs) or slow onset/lack of efficacy. The endogenous opioid system is involved in stress and emotion regulatory processes and its role in MDD has been implicated. Although several KOR antagonists including JDTic and PF-04455242 were discontinued in early clinical trials, ALKS 5461 and CERC-501(LY-2456302) survived and entered into Phase-III and Phase-II trials, respectively. Considering the efficacy and safety of early off-label use of buprenorphine in the management of the treatment-resistant depression (TRD), it will be not surprising to predict the potential success of ALKS 5461 (a combination of buprenorphine and ALKS-33) in the near future. Moreover, CERC-501 will be expected to be available as monotherapy or adjuvant therapy with other first-line antidepressants in the treatment of TRD, if ongoing clinical trials continue to provide positive benefit-risk profiles. Emerging new researches might bring more drug candidates targeting the endogenous opioid system to clinical trials to address current challenges in MDD treatment in clinical practice.

## 1. Brief Introduction of Major Depressive Disorder

Unlike other depressed mood which might be a normal reaction to life events, major depressive disorder (MDD) is a psychotic disorder, with chief manifestations of depressed mood, generalized anxiety, irritability or guilt. Besides these negative emotions, MDD is also associated with changes in physical, social and intellectual functions, resulting in a significant incapability or disability. In severe conditions, patients may experience delusions, hallucinations, suicidal ideation or behavior.

According to World Health Organization (WHO), it has been estimated that more than 350 million people were affected by depression.^[1]^ In the United States, major depression has a point prevalence of approximately 3 to 5% in males and 8 to 10% in females. Lifetime prevalence is up to 10% in males and 20 to 25% in females.^[2]^ There were no formal epidemiological studies on MDD conducted in China. However, a meta-analysis^[3]^ demonstrated that the overall estimation of current, 12-month and lifetime prevalence of MDD was 1.6, 2.3, 3.3 % respectively in Mainland China. Moreover, the World Mental Health Survey Initiative from 18 high and low- to middle-income countries found that the 12-month prevalence of Major Depressive Episodes (MDE) was 3.8% in China, similar to that in high-income countries. But the lifetime prevalence of MDE ^[4]^ was somewhat lower in China (12.0%) than in high-income countries (28.1%). Thus, MDD represents a major threat to both developed and developing countries worldwide.

## 2. Current Antidepressants with Clinical Relevance

Tricyclic antidepressants (TCAs) were firstly introduced during the era of explosive birth of psychopharmacology in 1950s. These agents exert their anti-depressive effects mainly by blocking the serotonin transporter (SERT) and the norepinephrine transporter (NET). However, due to the promiscuous nature of pharmacology, there are additional anticholinergic, antihistaminic and antipsychotic components^[5]^ of most TCAs, which are associated with respiratory depression, seizures, urinary retention and cardiovascular toxicities. To date, TCAs, such as imipramine **(1)** ^[6]^, clomipramine **(2)** ^[7–10]^ and amitriptyline **(3)** ^[11, 12]^ are recommended as the multi-line therapies for patients with major depression.

MAOIs (monoamine oxidase inhibitors, e.g., isocarboxazid **(4)**,^[13–15]^ moclobemide **(5)**,^[16–18]^ and toloxatone **(6)** ^[19]^) represent another category of anti-depressants.^[20, 21]^ These agents inhibit the activities of monoamine oxidases, which are responsible for oxidation and inactivation of monoamine neurotransmitters (e.g., serotonin, dopamine and norepinephrine). The potential interaction between MAOIs and tyramine-enriched food might lead to severe or fatal hypertension.^[22, 23]^ Moreover, since monoamine oxidases bear similar structures and functions to cytochrome P450 enzymes, most MAOs were also identified as drug-metabolizing enzymes and could induce potential drug-drug interactions. Today, MAOIs have been indicated for the treatment of depression when other treatments failed to work. ^[24]^


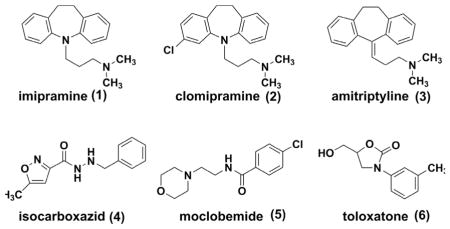


The availability of selective serotonin reuptake inhibitors (SSRIs) in 1980s changed the management of MDD significantly. ^[25]^ Citalopram ^[26, 27]^ (as well as the *S*-enantiomer escitalopram **(7)**, ^[28–30]^) sertraline **(8)** ^[31–33]^ and fluoxetine **(9)** ^[34]^ have become the most prescribed antidepressants in many countries. SSRIs are effective, more tolerable and safer over TCAs or MAOIs. Introduction of a norepinephrine component yielded serotonin–norepinephrine reuptake inhibitors (SNRIs), such as venlafaxine **(10)**, ^[35–37]^ duloxetine **(11)**, ^[38, 39]^ milnacipran **(12)**.^[40, 41]^ These agents are believed to deliver similar potent anti-depressant activities and also to be effective for the treatment of neuropathies. ^[42, 43]^


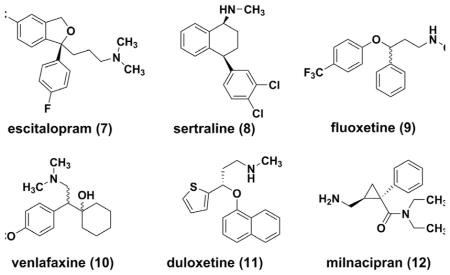


Both SSRIs and SNRIs are the most used first-line anti-depressants worldwide. However, some challenges remain unresolved. Patients with MDD might not benefit from SSRIs until after 2–4 weeks of treatment. Such slow-onset places a heavy emotional and psychological burden on patients with MDD.^[44, 45]^ Many researchers are expediting the discovery of agents with inhibitory activities against multiple monoamine reuptake, such as SNDRIs, or triple re-uptake inhibitors, aiming to avoid prolonging the treatment phase before attainment of optimal efficacy expectation^[46]^. But too much dopamine activity might have dose-limiting factors due to its stimulant effect. Currently, no triple re-uptake inhibitors have been marketed to date, but a few candidates have already entered into clinical trials (e.g., LY03005, EB1010, SEP-225289). Moreover, approximately 30–40% patients complained of lack of efficacy during the treatments of SSRIs or SNRIs, which represents a major unmet medical need. ^[47–49]^ Patients who responded poorly have to turn to less preferred treatments with a combination of multiple drugs. In October 2004, FDA required manufacturers of antidepressant medications to add a black-box warning that these medications, specifically fluoxetine, sertraline and other SSRIs, may increase suicidality in children and adolescents less than 24 years old, as indicated in studies of MDD and other psychiatric disorders. ^[50–53]^ This warning reduced the use of SSRIs or SNRIs for pediatric patients with MDD.

While the monoamine deficiency hypothesis is undoubtedly successful, leading to the successful development of MAOIs as antidepressants, MAOIs only relieves some of the patients with MDD. Current research is focused on the identification of novel targets and relevant ligands to resolve the unmet need as depicted above.

## 3. Kappa Opioid Receptor (KOR) as a Druggable Target for Depression

The association between the endogenous opioid system and mood regulation has been well established. ^[54]^ Most opioids with clinical relevance are practically Mu opioid receptor (MOR) agonists, which are effective for most of the severe pain. MOR agonism is associated with analgesia, euphoria and mood changes, as well as dose-limiting side effects, such as constipation, respiratory depression and addiction. In contrast, KOR is another member of the opioid receptor family, whose agonism results in as analgesia, dysphoria, hallucination, and psychotomimetic effects. ^[55, 56]^The endogenous dynorphin-KOR system is now being extensively investigated in rodent models for its role in depression to explore the potential use of KOR antagonist in the treatment of depression ^[57–61]^. KOR has been proposed to be involve in the stress system implicated in depression pathophysiology and was identified as an important component in depression and other psychiatric disorders characterized by reward dysfunction.^[58, 59, 62]^ The development of KOR antagonists as potential antidepressants are mainly driven ^[63]^ by their antidepressant-like effects in *in vivo* studies.^[64]^ The prototype of non-peptide KOR antagonist, nor-BNI, could produce antidepressant-like effects in both forced-swimming (FS) ^[65]^ and learned helplessness (LH) ^[66]^ assays in rodent models. Other selective KOR antagonists (e.g. JDTic) also showed antidepressant-like effects *in vivo.* ^[67, 68]^ Moreover, the pan opioid antagonist naloxone against all opioid receptors was also found to have antidepressant effect in the learned helplessness (LH) model. ^[69]^

## 4. Opioid Receptor Antagonists as Potential Antidepressants

Early interest for KOR ligands were stemmed from the discovery of U50488 ^[70]^ in the 1980s, an arylacetamide without the opiate motif but with highly selective KOR agonistic activities. With U50488 as the prototype of kappa full agonists, a large family of related compounds have been synthesized and a few (e.g., spiradoline, enadoline) candidates were brought to clinical trials in 1990s. However, the clinical trials were terminated early due to the dose-limiting toxicities of dysphoria, hallucinations and psychotomimetic effects. ^[71, 72]^ In contrast, KOR antagonists were initially developed as pharmacological tools to characterize KOR and its agonists, but their potential clinical use as potential antidepressants were not addressed until recently. ^[64]^

### 4.1 Opiate Derived KOR Antagonists

#### Nor-binaltorphimine (13)

Nor-binaltorphimine (nor-BNI) was described as a highly selective and potent non-peptide KOR antagonist in late 1980s. ^[73]^ It is a bivalent dimer of naltrexone *via* a pyrrole ring in its structure.^[74]^nor-BNI demonstrated a high affinity to KOR (K_i_ =0.26nM) in guinea pig brain.^[75]^ While in guinea pig ileal (GPI) longitudinal muscle preparations, the antagonistic potency of this compound was determined to be 0.41nM for the KORs ^[76]^, with approximately 170 and 150 times more potency than for mu and delta opioid receptors (DOR), respectively.^[77]^ For pharmacokinetic characteristics, nor-BNI at a dose of 20 mg/kg, s.c. demonstrated a biphasic elimination pattern in mice, with the rapid phase for 0.75–4 hours and the slow phase for 4–48 hours respectively.^[78]^ Pharmacodynamically, the extremely long-acting mechanism of nor-BNI was shown in the blocking of the analgesic effect induced by U69,593 and bremazocine for up to 504 hours *in vivo*. ^[79]^ As a consequence, there are significant concerns of drug accumulation for developing non-BNI as a clinical medication.


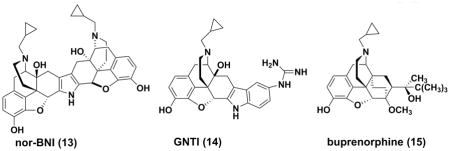


#### GNTI (14)

5′-guanidinonaltrindole (GNTI) was synthesized and characterized by Portoghese and coworkers from the highly selective DOR antagonist naltrindole.^[80]^ GNTI is also a natrexone derivative, but it is structurally dissimilar to nor-BNI. Later Structure-activity Relationship (SAR) analysis demonstrated that both compounds bear similar structural loci for KOR selectivity, especially for the relatively conserved basic component, which was confirmed by site mutatgenesis studies.^[63]^ GNTI has a K*i* value of 0.14nM for KOR transiently expressed in rat HEK-293 cells [Ki ratio: MOR/KOR=712 DOR/KOR=177],^[81]^ with an approximate four-fold increase compared to nor-BNI. It also demonstrates high KOR antagonistic activities (K_e_=0.16nM) in Guinea-pig ileum (GPI) preparations. By intramuscular administration, GNTI could reverse the effects of the KOR selective agonist U50, 488 on rhesus monkeys dose- and time-dependently, and its pharmacokinetics is characterized by a slow onset and long duration of action, with its antagonistic effect peaking after 24 hours.^[82]^ However, GNTI is orally inactive probably due to its poor blood–brain barrier (BBB) penetration as the consequence of a fully ionized guanidinium group in its structure. ^[83]^


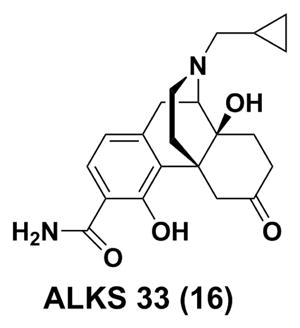


#### Buprenorphine (15)

Buprenorphine is a semisynthetic opioid derived from the opiate alkaloid thebaine. It was initially developed as a long acting analgesic for chronic pains^[84]^ and substitution treatment for opioid addiction.^[85–87]^ Due to its unique KOR antagonistic and MOR partial agonistic activities, the anti-depression potential of buprenorphine has been investigated extensively in animal models ^[88]^ and clinical trials.^[86, 87, 89]^ An early open label study in patients with treatment-refractory, unipolar, nonpsychotic, major depression, suggested a possible role of buprenorphine in the treatment of refractory depression.^[90]^ Low-dose buprenorphine may be a novel medication that provides a rapid and sustained improvement for older adults with treatment-resistant depression.^[91]^ Despite of these encouraging results, there is a mu opioid component involved in the pharmacological profile of buprenorphine, potentially resulting in opioid-like side effects, such as nausea, constipation and dyspnea.^[92, 93]^

ALKS 5461, a fixed combination of buprenorphine and ALKS 33 (samidorphan, **16**) for sublingual administration, has been developed by Alkermes as a potential treatment for patients with MDD not responding to SSRIs or SNRIs. ALKS 33 is a full MOR antagonist, which was employed to reverse the known side effects induced by the Mu opioid component of buprenorphine. In a randomized, double-blind, placebo-controlled phase II study in subjects with major depressive disorder (ClinicalTrials.gov Identifier: NCT01500200), ALKS 5461 demonstrated evidence of efficacy in patients with MDD not responding to SSRIs or SNRIs. Moreover, a substantial effect was attained after treatment for seven days. ALKS-5461 was granted Fast Track Designation by the Food and Drug Administration (FDA) for treatment-resistant depression in October 2013. Phase III trials were initiated in 2014 and the preliminary results were favorable. If ALKS 5461 were to be authorized successfully in late 2016 or 2017, it might meet some medical need for patients inadequately controlled by SSRI and SNRI monotherapy.


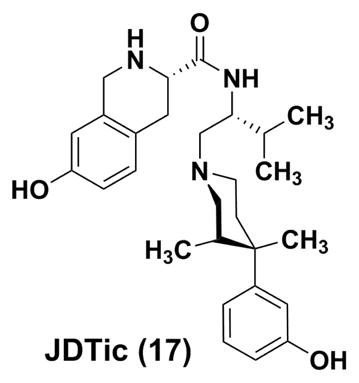


### 4.2 JDTic (17)

With pethidine (also known as meperidine) as the prototype, the synthetic opioid analgesic of the phenylpiperidine class has a relatively long history of clinical use. In 1978, Zimmerman and co-workers described that introduction of a unique (3R, 4R)-dimethyl substitution results in (*3R, 4R*)-Dimethyl-4-(3-hydroxyphenyl)-piperidines (e.g., LY272922) as non-selective opioid antagonists ^[94]^. Based on the structure of JPP-6 with KOR binding other than functional selectivity,^[95]^ Carroll and co-workers at Research Triangle Institute (RTI International) discovered JDTic ^[96]^ to be a potent, long and orally-active selective KOR antagonist, although it was later considered that neither the 3-methyl nor 3, 4-dimethyl groups present in JDTic and analogs are required to produce potent and selective KOR antagonists. ^[97]^

Besides its high KOR binding affinity (Ki=0.32 nM) in guinea pig brain, the extremely high antagonism and selectivity over other opioid receptors of JDTic was confirmed in agonist stimulated [^35^S]GTP-γ-S Binding assay of Cloned Opioid Receptors (K*_e_* = 0.006 nM, MOR/KOR ratio=570, DOR/KOR ratio > 16600). JDTic could antagonize the antinociceptive effects of the KOR agonist U50, 488H, but had no effect on morphine-induced behaviors in mice. In U50, 488-induced diuresis rat test, JDTic, suppressed diuretic activity with a greater potency than that of nor-BNI ^[98]^. The psychiatric effects of JDTic were also investigated in a number of rodent models, such as nicotine reward, ^[99]^ alcohol seeking and withdrawal anxiety,^[100, 101]^ as well as opiate abuse,^[102]^ and promising findings were observed. After intraperitoneal injections in mice, the brain concentration of JDTic peaked within 30 minutes and decreased gradually over a week, despite a mechanism of P-gp-mediated efflux ^[83]^. The high apparent volume of distribution and slow elimination from the brain for JDTic suggest a very high tissue affinity. ^[83]^

The first human trial of JDTic was halted abruptly in May 2012 due to apparent clinical toxicity (non-sustained ventricular tachycardia (NSVT)).^[103]^ However, this cardiotoxicity was not observed in both *in vitro* and *in vivo* assays before human studies. Further investigation is needed to determine whether observed cardiac involvement is specific to JDTic or possibly represents a class effect of KOR antagonists.^[104]^

### 4.3 KOR Antagonists from Pharma R&D Pipeline CERC-501 (18)

CERC-501 (formerly known as **LY-2456302**) ^[105]^ which was originally developed by Eli Lilly and later by Cerecor, is a non-peptide, short-acting KOR-selective antagonist (K_i_=0.813 nM, MOR/KOR=21 DOR/KOR=135). This compound demonstrates a >30-fold functional KOR selectivity over MORs and DORs. Oral administration of CERC-501 could reverse the analgesia induced by U69593 (1 mg/kg, sc) in rat formalin assays (ED_50_=0.4 mg/kg), whereas no reversal effects were observed after 1-week of pre-treatment ^[105]^. In mouse forced swim assays, 10 mg/kg oral dose of CERC-501 was found to reduce immobility responses similarly to an optimal dose of the TCA imipramine (15 mg/kg, ip). A synergic effect was also observed for CERC-501 (3 mg/kg, po) and citalopram (3 mg/kg, ip) in combination, thus suggesting that CERC-501 may augment antidepressant efficacy in patients who do not achieve adequate improvement of depressive symptoms on standard MAOI medications. ^[105]^ CERC-501 has rapid absorption (t_max_: 1–2 h) and good oral bioavailability (F = 25%). ^[106]^


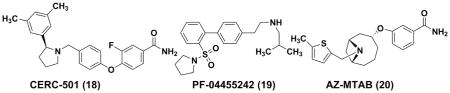


In clinical trials, CERC-501 displayed rapid oral absorption and a terminal half-life of approximately 30–40 hours in healthy subjects ^[107]^. Plasma exposure of CERC-501 increased proportionally with increasing doses and reached steady state after 6–8 days for once-daily dosing ^[107]^. A proof-of-concept trial of CERC-501 for augmentation of antidepressant therapy in treatment-resistant depression was initiated in April 2015, and the results are estimated to be available in December 2016.

#### PF-04455242 (19)

PF-04455242, developed by Pfizer, is a novel KOR antagonist from biphenyl derivatives. It demonstrates a high affinity for human KOR (Ki=3 nM), with approximate 20-fold selectivity over MOR (MORs; Ki = 64 nM) and negligible affinity for DOR receptors (Ki > 4 μM) ^[108]^. In mouse tail-flick assay, the analgesia induced by spiradoline (5.6 mg/kg s.c., KOR agonist) and morphine (3.2 mg/kg s.c., MOR agonist) could be reversed by PF-04455242 in a dose dependent pattern, with ID_50_ values of 1.5 and 9.8 mg/kg, respectively. Moreover, PF-04455242 was as effective as nor-BNI in rodent models of depression, such as mouse forced-swim test and mouse social defeat stress assay.^[108]^

Although its brain penetration capability and potential hERG profile which was associated cardiotoxicity are acceptable, PF-04455242 has some binding affinities for 19 out of 120 off-targets (the proteins targets other than therapeutic purposes ^[105]^, posing relevant safety concerns and potential side effects. Furthermore, an KOR occupancy phase-I clinical trial of PF-04455242 was terminated on January 6, 2010, due to toxicology findings in animals exposed to PF-04455242 for three months [ClinicalTrials.gov Identifier: NCT00939887].

#### AZ-MTAB (20)

AZ-MTAB, an 8-azabicyclo [3.2.1] octan-3-yloxy-benzamide, is a selective KOR antagonist developed by AstraZeneca.^[109, 110]^ As shown in the human [^35^S] GTPγS assay with Dynorphin A(1–13) as the agonist, the KOR antagonistic activity of AZ-MTAB was determined to be 20nM, which showed approximately 30- and 400-fold selectivity over MOR and DOR antagonism, respectively.^[110]^ Besides having relatively favorable pharmacokinetic profiles, it could reverse KOR agonist induced diuresis in rats.^[105]^ However, due to its relatively high hERG activity (IC_50_=0.26 μM) and the overall hERG liability of this structure class,^[105]^ AZ-MTAB or any structurally related candidates could not enter clinical trials.

## 5. Conclusion

There are currently multiple drug classes available for the treatment of major depressive disorder, including TCAs, MAOIs and SSRIs, as well as SNRIs. However, approximately 30–40% patients with MDD fail to benefit from these medications, especially for those with treatment-resistant depression responding poorly to at least two antidepressants. The emergence of KOR antagonists seems promisingly to satisfy this unmet medical need. In fact, the efficacy and safety of early off-label use of buprenorphine has been promising in the management of treatment-resistant depression. It seems that ALKS 5461 (a combination of buprenorphine and ALKS-33) is hopeful in entering clinical practice in the near future. Moreover, in consideration of current pre-clinical and clinical evidences, CERC-501 (LY-2456302) will be expected to be available as monotherapy or adjuvant therapy with other first-line antidepressants in the management of treatment-resistant depression, if ongoing clinical trials continue to provide positive benefit-risk profiles. Emerging new research interests might bring more useful drug candidates to clinical trials to address the current challenges in MDD treatment in clinical practice.
